# Feasibility of delivering TeleCHAT: A comprehensive high-dose aphasia treatment via telerehabilitation

**DOI:** 10.1177/02692155251375667

**Published:** 2025-09-26

**Authors:** Genevieve Vuong, Jade Dignam, Clare Burns, David Copland, Hannah Wedley, Katherine O’Brien, Annie J Hill

**Affiliations:** 1 Queensland Aphasia Research Centre, 95357 The University of Queensland, Brisbane, QLD, Australia; 2 Faculty of Health and Behavioural Sciences, School of Health and Rehabilitation Sciences, 1974 The University of Queensland, Brisbane, QLD, Australia; 3 Centre for Research Excellence in Aphasia Recovery and Rehabilitation, 2080 La Trobe University, Melbourne, VIC, Australia; 4 Surgical Treatment and Rehabilitation Service (STARS) Education and Research Alliance, The University of Queensland and Metro North Health, Brisbane, QLD, Australia; 5 Faculty of Health, 4571 Southern Cross University, Bilinga, QLD, Australia; 6 Speech Pathology and Audiology Department, Royal Brisbane and Women's Hospital, Metro North Health, Brisbane, QLD, Australia

**Keywords:** Aphasia, telerehabilitation, intensive comprehensive aphasia programme, feasibility, tailored support

## Abstract

**Objective:**

To evaluate the feasibility of delivering 50 h of comprehensive, high-dose aphasia treatment via telerehabilitation (TeleCHAT) to people with aphasia and their support people.

**Design:**

A non-randomised one-armed quasi-experimental pre-post feasibility study.

**Setting:**

TeleCHAT was delivered from dedicated tele-suites in university spaces within a tertiary hospital. Participants received therapy in their homes via telerehabilitation using a configured telerehabilitation system which used videoconferencing software Zoom^®^.

**Participants:**

Three cohorts of people with aphasia (*n* = 12), support people (*n* = 11), and speech-language pathologists (*n* = 2) participated.

**Intervention:**

Participants completed technology training, goal setting, and clinical treatment planning prior to the intervention. The TeleCHAT intervention included 50 h of goal-directed aphasia therapy, delivered 3–5 days per week over 8 weeks.

**Main measures:**

Mixed-methods data was collected on participant demographics, aphasia profiles, achievement of dose, comprehensiveness of therapy, and support people participation.

**Results:**

A diverse group of people with aphasia completed TeleCHAT. Nine participants received the intended dose of 50 h, with the remaining three closely approaching dose. A high proportion of sessions were spent actively engaged in therapeutic tasks (94–100%). A comprehensive array of 42 therapy activities was delivered and tailored to goals across the International Classification of Functioning, Disability and Health Framework. All participants had a support person participate actively in at least one session.

**Conclusions:**

It was feasible to deliver the core components of the TeleCHAT programme via telerehabilitation. As intended, a heterogeneous group of people with aphasia received a high-dose of tailored, comprehensive aphasia therapy, with the active participation of support people.

## Introduction

Post-stroke aphasia is an acquired language impairment impacting all domains of the International Classification of Functioning, Disability and Health Framework.^1^ Intensive comprehensive aphasia programmes (ICAPs) deliver individual and group therapies, along with patient and family education, targeting all domains of functioning.^2^ Due to the breadth of therapies involved in an ICAP, speech pathologists require specialised training, knowledge, and skills to deliver the programme. These programmes are delivered intensively (a minimum of 3 h/day for 5 days/week for 2 weeks) in a cohort model.^2^ Modified-ICAPs (m-ICAPs) is a term given to ICAPs that have modified one core element of the definition such as intensity or comprehensiveness.^3^

Evidence supports the clinical efficacy and psychosocial benefits of ICAPs,^4–7^ with one randomised controlled trial reporting better outcomes on aphasia-related quality of life and caregiver burden than usual care.^8^ However, there are a limited number of ICAPs operating worldwide, with only 21 ICAPs and m-ICAPs identified across eight countries in 2021.^3^ Barriers such as transport access, patient mobility, fatigue, and limited family availability hinder participation in these programmes, especially for rural residents.^9–11^ Delivering ICAPs via telerehabilitation may alleviate these barriers, enabling better access to this specialised service for people with aphasia.

It is feasible to deliver individual aphasia therapies via telerehabilitation.^12,13^ However, it has been suggested that the telerehabilitation delivery of an ICAP may present challenges, including assessing participant readiness, scheduling logistics, adapting treatment for more severe aphasia, and maintaining comprehensive therapy.^3^ While no known studies have reported on the feasibility of delivering an ICAP via telerehabilitation, a system has been configured to deliver one such programme, comprehensive high-dose aphasia treatment (CHAT) via telerehabilitation (TeleCHAT).^14^ This study aimed to evaluate the feasibility of delivering TeleCHAT, that is, whether the configured system allowed for the delivery the core components of the TeleCHAT programme (completion of the programme by people with aphasia; high-dose therapy; comprehensive, tailored therapy; and active participation of support people) as intended in a research context.

## Methods

### Design

This prospective feasibility study employed a non-randomised one-armed quasi-experimental pre-post design. The study was conducted at the Queensland Aphasia Research Centre, located at the Surgical Treatment and Rehabilitation Service (STARS), Brisbane, Australia. TeleCHAT was delivered from 2021 to 2022 to three consecutive participant cohorts in an iterative manner, where data from each cohort was used to inform and modify the TeleCHAT programme for the next cohort. Ethical approval to conduct this research was received (HREC/2020/QRBW/61636 and 2020/HE002118).

### Participants

Three participant groups were recruited: people with aphasia, support people, and speech-language pathologists. People with aphasia were eligible if they were at least 1-month post-stroke, aged over 18 years, had aphasia as identified by a speech-language pathologist using the Language Screening Test^15^ or the Comprehensive Aphasia Test (CAT),^16^ had access to videoconferencing compatible devices with internet connectivity at their residence (e.g. desktop computer, laptop, or tablet), and had basic premorbid English-language proficiency. People were excluded if they had co-morbidities that would prevent participation in usual aphasia rehabilitation (e.g. severe cognitive impairments, profound hearing or visual impairment, medical instability). People were included if they had co-morbidities that did not directly prevent participation in language-based rehabilitation, such as motor-speech disorders or limb paresis. Participants were required to cease usual speech pathology treatment for aphasia for the duration of the intervention. Support people were family members or friends who were invited to participate by the person with aphasia. Eligible support people were aged over 18 years and had basic English-language proficiency. Two qualified speech-language pathologists participated in the study and delivered the TeleCHAT programme to people with aphasia across all three cohorts. They both had graduate experience (0.5- and 5-years’ experience respectively) in delivering aphasia rehabilitation, and both had minimal experience in delivering speech pathology intervention in individual formats via synchronous telerehabilitation.

**Figure 1.**
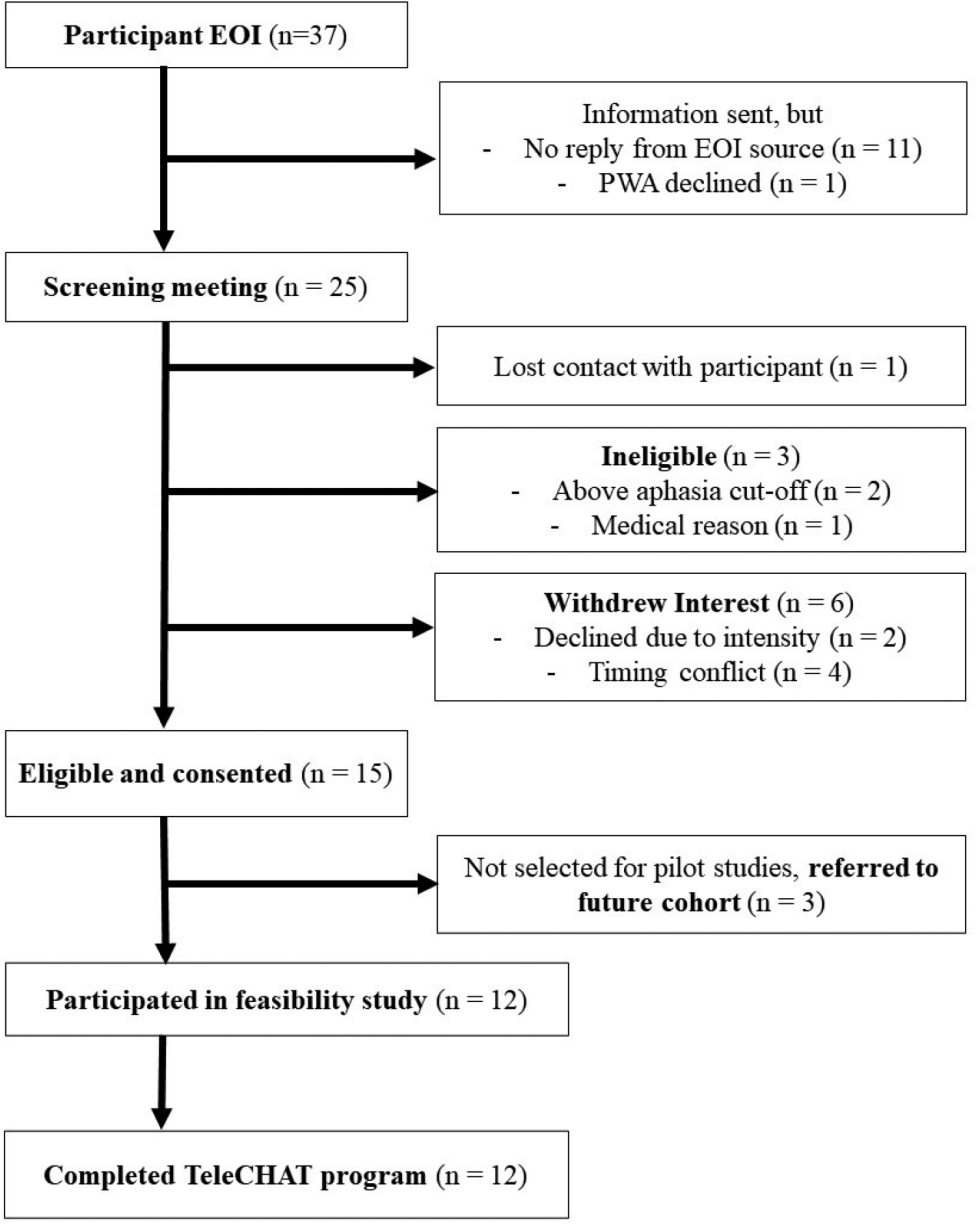
Flowchart outlining the recruitment of people with aphasia to TeleCHAT. TeleCHAT: comprehensive, high-dose aphasia treatment via telerehabilitation; EOI, expression of interest; PWA, person/s with aphasia.

Recruitment of people with aphasia and their support people (Figure 1) occurred from April 2021 to November 2022 via clinical and professional networks. Twelve participants were selected against sampling criteria and consented. This sample size was chosen as it allowed for three cohorts of TeleCHAT (four people with aphasia per cohort) to be delivered and refinements to occur within a 20-month period. Purposeful maximum variation sampling was used to select a heterogeneous group of participants reflective of the aphasia population and to increase generalisability.^17^ Effort was made to include participants ranging in age, gender, time post-stroke, geographical location, aphasia profile and severity, and technology confidence.

### Baseline assessment, goal setting, planning, and training

**Figure 2.**
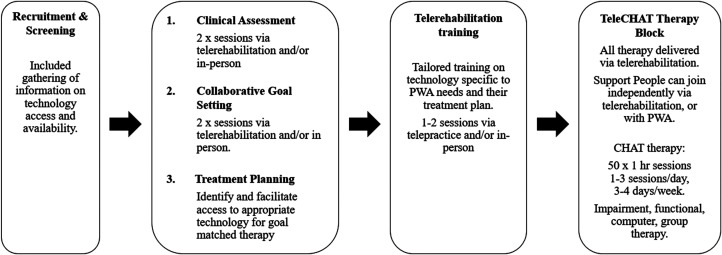
TeleCHAT programme outline. Note. This outline specifies key components of the TeleCHAT programme and telerehabilitation-specific additions/modifications to the CHAT programme described in Dignam et al. (2023)^5^. All parts of the TeleCHAT programme were conducted collaboratively with people with aphasia, their families and/or support people, and the speech-language pathologist, except for treatment planning, which was conducted between the clinicians and the research team. TeleCHAT: comprehensive, high-dose aphasia treatment via telerehabilitation; PWA: people with aphasia.

Baseline clinical assessment, collaborative goal setting, and telerehabilitation training were conducted across multiple sessions via telerehabilitation and/or during home visits to inform personalised therapy planning (Figure 2) (see Supplemental File 1 for a full description of this process). One participant completed assessment and training entirely via telerehabilitation. Evaluation of the person with aphasia's language skills was conducted using the CAT,^16^ which was translated for telerehabilitation delivery by Pitt.^18^ Baseline assessment also included evaluation of the person with aphasia's internet connection and baseline technology skills and confidence. Speech-language pathologists considered how the individually planned therapy would be administered via telerehabilitation using the person with aphasia's personal devices and, where necessary, identified additional technical solutions to ensure the person with aphasia could participate and interact. Where a participant did not have the necessary device, this equipment was lent to participants.

Training packages were developed and provided to all participant groups to support their participation in TeleCHAT (see Supplemental File 1 for full details of the training). The speech-language pathologists were provided training on the delivery of ICAPs and telerehabilitation prior to commencing recruitment of participants to TeleCHAT. The first cohort of people with aphasia and their support people received training 3–4 weeks prior to intervention. Training for participants with aphasia incorporated practical sessions, where participants learnt how to use the telerehabilitation technology. Following feedback from the first cohort, the training was revised. The timing of training was moved to 1 week before commencement of therapy, to optimise retention of technical skills and provide time for the speech-language pathologists to tailor training to the individuals’ therapy plans. Furthermore, to enhance the applicability and practicality of the telerehabilitation training, a mock telerehabilitation activity was added, whereby a speech-language pathologist was present in person with aphasia's home while the other speech-language pathologist delivered the activity via telerehabilitation from the Queensland Aphasia Research Centre.

### Intervention

The TeleCHAT programme replicated the CHAT programme protocol^5^ with minor modifications to allow for delivery via telerehabilitation (Figure 2). A full description of the TeleCHAT programme is provided using the telehealth extension of the Template for Intervention Description and Replication checklist to enable replicability in future studies (TIDieR-Telehealth)^19^ (Supplemental Table 1). The description also includes all elements from the Extended TIDieR framework for speech and language pathology interventions for aphasia after stroke.^20^

The intervention consisted of 50 h of evidence-based, goal-directed aphasia therapy. Therapy includes 14 h each of impairment, functional, and computer-based therapy, and 8 h of psychosocial group therapy, delivered over 8 weeks.

The TeleCHAT programme was delivered using a configured telerehabilitation system,^14^ including the enterprise version of the videoconferencing platform Zoom^®^ (refer to Supplemental Table 1 for further details of the system). The person with aphasia and their support person used an internet-enabled device (e.g. computer/laptop/iPad) with an in-built or external microphone and speakers to join the remote therapy session. The technology system was tailored to the participant's treatment goals, their language and physical abilities, technology confidence and skill, as well as their available personal hardware and devices. If participants’ therapy plans involved mobile applications or secondary display of material (e.g. handwriting), then secondary devices (such as an iPad, Android tablet, or smartphone) were used. The speech-language pathologists conducted the TeleCHAT sessions from dedicated telerehabilitation suites at the Queensland Aphasia Research Centre. These telerehabilitation suites were optimised for audio and visual clarity and clinicians had access to double screens and a document camera. During COVID-19 lockdown periods, speech-language pathologists delivered TeleCHAT sessions from a private room at their residence. From their residence, they continued to have access to the same technology, except for a document camera.

### Outcome measures and data analysis

The outcome measures used to evaluate the feasibility of TeleCHAT, as defined by the criteria of an ICAP and the CHAT protocol,^5^ are described as follows:

1. *Completion of the TeleCHAT programme by intended users*. This was measured by examining the percentage of participants who finished the TeleCHAT programme and exploring their demographics. Participant demographics and baseline technical confidence were collected on a pre-intervention survey. Participants’ aphasia profiles were derived from pre-therapy language assessment on the CAT.^16^ Participant completion rates in therapy sessions were collected from therapy attendance logs completed by the speech-language pathologist.
2. *Dose of therapy*. This was assessed using the dose dimensions of the multidimensional dose articulation framework.^21^ Data was extracted from therapy attendance logs on session duration, intervention days, number and spacing of sessions, session length, and session density. Session density was defined as the proportion of session length spent actively engaged in therapeutic tasks (active episodes). In this study, inactive episodes were defined as pauses or interruptions caused by technical issues and user difficulties. Reasons for the occurrence of inactive episodes and session cancellations were logged after each session by the speech-language pathologist, in an ‘Issue-registration form’ adapted from Øra et al.^22^
3. *The comprehensiveness of tailored therapy*. The tailoring of therapy to participant goals and the range of therapy tasks delivered to each person with aphasia were extracted from the therapy plans and session notes. Any impacts on therapy delivery due to issues related to the telerehabilitation system were recorded on issue-registration logs. Activities significantly impacted by inactive episodes (i.e. where inactive episodes lasted >6 min; 10% of intended session length) were collated to identify any inefficiently delivered therapy activities.
4. *The active participation of support people in TeleCHAT*. Active participation was defined as whether support people attended collaborative goal setting and/or >1 therapy session that provided education and/or training related to aphasia and its management. Support person attendance was recorded in session notes. The level of technical assistance support people provided to people with aphasia was recorded by speech-language pathologists each session, using a 5-point Likert scale (0 = No help at all, 1 = Help once or twice, 2 = Help on some tasks, 3 = help on most tasks, 4 = help on all tasks).

Quantitative data, including completion and attendance rates, dose, and survey ratings, were analysed using descriptive statistics (mean, range, standard deviation, and frequency). The therapy goals and activities were analysed qualitatively through categorisation into groups based on their language and domains of functioning.

## Results

### Completion of the TeleCHAT programme by intended users

A maximum variation sample that represented a large majority of the aphasia population was successfully recruited. The participants had varying demographics, aphasia profiles, technological confidence, and technology devices (Table 1; refer to Supplemental Table 2 for individual demographics). All 12 people with aphasia (100% of participants) completed the TeleCHAT programme. The seven males and five females were aged between 24 and 81 years (*M* = 65.2, *SD* = 15.3) and lived in rural (*n* = 1), regional (*n* = 4), and metropolitan (*n* = 7) locations. Participants were 4–78 months post-stroke onset (*M* = 26.9 months; *SD* = 25.9 months). Participants presented with a range of aphasia severities across various language domains, as measured by the CAT modality mean (*M* = 50.7, *SD* = 5.9, range = 40.2–58) (Supplemental Table 3). All but one participant had previously received speech therapy via telerehabilitation and/or participated in videoconference calls, however, on average, they rated themselves to have less than moderate confidence using technology (*M* = 2.7, *SD* = 1.4, range = 1–5).

**Table 1.** Group-level participant demographics and technology characteristics.

Parameter	*n*	Mean	*SD*	Range
Sex		n/a	n/a	n/a
Male	7			
Female	5			
Age (year range)		65.17	14.6	24–81
24–65	4			
>65	8			
International standard classification of education^a^		3.7	1.8	1–7
1–2	3			
3–4	6			
5–7	3			
Handedness		n/a	n/a	n/a
Right	11			
Left	1			
Time post-stroke onset (months)		26.9	24.8	4–78
<6	2			
6–12	3			
12–24	3			
>24	4			
Number of strokes		1.4	0.75	1–3
1	9			
2–3	3			
Lesion hemisphere of stroke		n/a	n/a	n/a
Right	8			
Left	1			
Both	1			
Comorbidities impacting^b^		n/a	n/a	n/a
Speech	6			
Voice	1			
Sight	3			
Upper limb movement	3			
Location^c^		n/a	n/a	n/a
Metropolitan	7			
Rural	4			
Remote	1			
Aphasia severity^d^		50.7	5.6	40.2–58
40–50	4			
50–55	5			
55–60	3			
Personal technology devices^b^		n/a	n/a	n/a
Laptop/computer	10			
iPad/Android tablet	11			
Phone	7			
Confidence using technology^e^		n/a	n/a	n/a
1–2	6			
3	2			
4–5	4			

a 1: primary, 3: upper secondary, 5: short cycle tertiary, 7: master's or equivalent level.

b May be attributed to more than one person.

c According to Rural, Remote, and Metropolitan Area (RRMA) classification.

d Average total of six tests on the comprehensive aphasia test *t*-scores.

e Rating on 5-point scale with 1 = Definitely no to 5 = Definitely, yes.

### Dose of therapy

Across the three cohorts (*n* = 12), 98.5% (591 sessions) of the 600 scheduled therapy sessions were successfully delivered, with only nine sessions cancelled and unable to be rescheduled. In total, 37 sessions were initially cancelled, three due to technology issues and 34 for patient- or clinician-related factors (e.g. illness, natural disaster, or scheduling conflict). Of these cancellations, 28 sessions (75.7%) were successfully rescheduled and completed.

A summary of the dose received by participants is reported using the multidimensional dose articulation framework^21^ in Table 2. Almost all participants (*n* = 11) attended therapy for 8 weeks. Overall, participants attended a median of 1–2 therapy sessions per day (range = 1–3 sessions/day of therapy), a median frequency of 4 days per week (range = 2–5 days/week). In terms of intensity, participants attended a median of six sessions per week (range = 4–8 sessions/week). Nine participants received the intended overall dose (sum of session length) of 50 h (range = 51 h 14 min–52 h 51 min), and the remaining three participants closely approached the intended dose (participant 7 = 49 h 17 min, participant 3 = 48 h 43 min, and participant 11 = 44 h 13 min). As indicated by session density, participants spent a high proportion of time (range = 94–100%) actively engaged in therapy activities. Hence, participants experienced a short duration of inactive episodes (time taken to resolve technical issues and user difficulties), ranging from a sum of 8 min to 3 h 12 min. Inactive episodes occurred in 36.2% of all delivered sessions (213 sessions); however, each of these interruptions only lasted a mean of 1 min (*SD* = 4 min, range = 0–29 min) and were resolved to enable session completion. Of the 628 sessions scheduled, only two were not delivered due to technical difficulties with either the internet connection or a device software update issue. The speech-language pathologists contacted two participants outside of session time to troubleshoot technical issues, but the issues were resolved by family members. One participant (participant 11) received a comparatively lower dose (duration = 7 weeks, sum of session length = 44 h 13 min) because they cancelled 1 week (7 h) of therapy due to a natural disaster. Therefore, participant 11's dose was considered an anomaly.

**Table 2.** Summary of overall mean and total dose per participant.

Person with aphasia	Duration (number of weeks)	Days of Intervention (range days/week)	Sessions (range sessions/day)	Sum of session length (h:min)	Sum of inactive episodes (h:min)	Sum of active episodes (h:min)	Session density^a^ (%)
1	8	4	1–2	52:51	0:54	51:57	98
2	8	4	1–2	52:45	0:34	52:10	99
3	8	3–5	1–2	48:43	0:08	48:34	100
4	8	4–5	1–2	52:47	3:12	49:35	94
5	8	2–4	1–3	52:49	2:25	50:24	95
6	8	3–4	1–2	51:35	0:42	50:52	99
7	8	4–5	1–2	49:17	3:09	46:07	94
8	8	3–5	1–2	51:22	2:43	48:38	95
9	8	4–5	1–2	51:14	0:38	50:35	99
10	8	3–4	1–2	51:31	1:07	50:24	98
11	7	5	1–2	44:13	1:29	42:44	97
12	8	3–5	1–2	51:51	1:06	50:45	98
Total	n/a	n/a	n/a	610:58	18:09	592:48	97
Mean	7.92	4.28	1.40	50:54	1:30	49:24	97
*SD*	0	0.63	0.41	3:44	1:04	2:39	71
Median	8	4	1.5	51:33	1:06	50:24	98
Range	7–8	2–5	1–3	44:13–52:51	0:08–3:12	42:44–52:10	94–100

*Note.* Dose was reported using the multidimensional dose articulation framework.^21^

Person with aphasia = Participant with Aphasia.

a Session density = (Sum Active Episodes/Sum Session Length) × 100.

Most participants received the intended dose for each therapy type delivered in TeleCHAT (i.e. 14 h each for impairment, functional, computer therapy and 8 h for group therapy) (refer to Supplemental Table 4 for individual dose data). The ratio of people who received the intended dose to people who received under the intended dose was 9:3 for impairment therapy, 10:2 for functional therapy, 10:2 for computer therapy, and 9:3 for group therapy. Participants who did not receive the full dose of a therapy type received between 9 and 68 min less than the intended dose (excluding participant 11).

### Comprehensiveness of tailored therapy

TeleCHAT therapy was successfully tailored to address the broad spectrum of participant goals. All participants engaged in collaborative goal setting resulting in communication goals that were distributed across domains of functioning (including body structure and function, activity, and participation, and environment) and language (spoken production, auditory comprehension, written production, and reading comprehension at word, sentence, and discourse levels), as exemplified in Table 3. Following clinical planning, each participant received a tailored treatment plan and technology set-up to address their personal communication goals.

**Table 3.** Examples of participant goals matched to the therapy activities delivered.

Language domain	Icf domain	Body structure and function	Activities, participation, and environmental factors	Body structure and function, activities, and participation	Activities and participation
	Exemplar goal	Impairment therapy	Functional therapy	Computer therapy	Group therapy
Spoken production	*I want to say core words (e.g. names, places and food) with/without use of photo aids, in conversations about my daily routine with family and close friends.*	Single word retrieval of core words (e.g. SFA/PCA, RIsupport people, RIPP) Mapping therapy with personalised phrases	Training use of multimodal communication and AAC software Script therapy Role play and practice with support person CPT Conversational coaching and problem solving with person with aphasia	Constant therapy – word retrieval, naming, simple sentence production activities StepByStep – naming and repetition Tactus and Tactus advanced – naming Training use of computer/apps for self-management	Practicing spoken production with other members in group
Reading comprehension	*I want to read and understand a chapter of a short book independently, or with visual aids/apps.*	ARCS with short generic paragraphs	Training use of text-to-speech technology ARCS with paragraph from personal book	Constant therapy – reading comprehension StepByStep – reading Tactus advanced reading ListenIn	Nil
Written production	*I want to text in a sentence to my friends and family about daily errands.*	CART for texting SFA/PCA with written answers VNeST with written answers	Training use of voice-to-text, and pre-saved texts Role play text conversation	Constant therapy – spelling StepByStep – spelling Tactus and Tactus advanced writing	Nil
Auditory comprehension	*I want to understand complex sentences when talking to my family, in a conversation.*	Sentence processing therapy – mapping therapy, VNeST, TUF)	CPT Script therapy of phrases to use to resolve comprehension breakdown	Constant therapy – AUDITORY comprehension ListenIn StepByStep – matching Tactus advanced comprehension	Education on CPT topic
Patient and family education	*I want to understand more about my stroke and aphasia and be able to educate others about it.*	Nil	CPT Script therapy of information to educate friends and family about aphasia Discussion of self-management of aphasia post therapy	Nil	Psychosocial therapy and education

AAC: augmentative and alternative communication; ARCS: attentive reading and constrained summarisation; CART: copy action retell therapy; CPT: communication partner training; Person with aphasia: person with aphasia; RIPP: repetition in presence of a picture; RIsupport people: repeated, increasingly speeded production; SFA/PCA: semantic feature analysis and phonological components analysis; TUF: treatment of underlying forms; VNeST: verb network strengthening treatment.

Across the three cohorts of TeleCHAT, 42 different aphasia therapy activities were delivered (refer to Supplemental Table 5 for a list of 37 individually delivered therapy activities). Therapy activities were categorised under the most relevant therapy approach, and their frequency of delivery counted (Table 4). Some therapy activities were delivered across multiple therapy types (e.g. Aphasia Scripts^23^ was used in computer therapy and functional therapy). The therapy approaches delivered most frequently in each therapy type included: impairment therapy targeting single-word retrieval of nouns and verbs (e.g. using combined semantic feature analysis and phonological components analysis^24^ (187 sessions across 11 participants), functional script therapy and role play with faded cues (109 sessions across 11 participants), and use of StepByStep^25^ software in computer therapy (87 sessions across 10 participants). Furthermore, communication partner training, discussion of self-management of aphasia post-therapy, and psychosocial group therapy and education were delivered to all 12 participants. Seven therapy activities involved an extended length of inactive episodes (Supplemental Table 6). Inactive episodes occurred most frequently and for the longest total amount of time for therapy programmes conducted on an iPad, Constant Therapy^26^ (9 sessions impacted for a total of 44 min) and Tactus Therapy^27^ (9 sessions impacted for a total of 64 min).

**Table 4.** Frequency of all therapy activities delivered across all therapy sessions.

*Participant with aphasia no.*	*1*	*2*	*3*	*4*	*5*	*6*	*7*	*8*	*9*	*10*	*11*	*12*	*Total frequency across all participants*	*Number of participants therapy delivered to*
*Aphasia severity* *^a^*	*47.7*	*55.7*	*54.2*	*46.0*	*55.7*	*40.2*	*58.0*	*51.8*	*40.7*	*54.0*	*50.2*	*54.2*	*-*	*-*
***Impairment therapy***														
*Narrative discourse therapy*	-	4	7	-	-	-	-	-	-	-	4	5	20	4
*Reading comprehension of paragraphs*	-	2	-	-	-	-	-	-	-	-	2	-	4	2
*Sentence processing and production therapy*	9	3	9	-	14	-	12	8	-	11	-	10	76	8
*Single word retrieval of nouns and verbs*	25	18	-	28	9	16	13	14	12	14	12	26	187	11
*Spelling therapy*	-	-	-	-	-	-	-	23	-	-	-	-	23	1
*Written sentence production*	-	-	-	-	-	-	-	-	-	13	-	-	13	1
***Functional therapy***														
*Communication partner training*	6	2	1	1	3	2	3	6	5	1	2	3	35	12
*Conversational coaching and problem solving for the person with aphasia*	-	2	-	1	7	-	2	-	1	4	2	6	25	8
*Discussion of self-management of aphasia post therapy*	5	6	4	9	7	9	9	6	4	4	5	4	72	12
*Multimodal communication and training of augmentative and assistive communication*	12	-	-	12	-	19	-	1	16	1	2	1	64	8
*Script therapy and role play with faded cues*	9	14	16	9	11	3	13	-	8	6	8	12	109	11
*Training use of technology to assist daily communication*	3	4	3	4	-	4	9	13	5	12	1	12	70	11
***Computer therapy***														
*Aphasia Scripts*	4	4	6	-	-	-	1	-	-	9	3	-	27	6
*Constant Therapy*	1	3	8	8	5	-	2	-	2	-	-	5	34	8
*Lingraphica TalkPath Therapy*	-	-	-	-	-	1	-	-	-	-	3	-	4	2
*ListenIn*	-	-	-	-	3	1	-	-	-	-	-	-	4	2
*StepByStep*	12	5	2	13	-	14	-	14	9	6	6	6	87	10
*Tactus Advanced therapy*	-	7	1	-	13	-	13	-	-	-	-	2	36	5
*Tactus Therapy*	-	-	-	-	-	-	-	-	5	4	3	3	15	4
***Group therapy***														
*Psychosocial therapy and education*	7	7	7	7	7	7	6	6	7	7	5	7	80	12

*Note*. Some tasks were delivered across multiple therapy types (impairment, functional, computer and group).

a Mean *t*-score across six modalities in the language battery of the Comprehensive Aphasia Test.^16^

### Active participation of support person in therapy

Each person with aphasia had at least one family member or friend participate in TeleCHAT sessions either in a separate location to the participant (*n* = 4), in the same room as the participant (*n* = 7), or a mix of both (*n* = 5). However, only 11 support people (eight females and three males, mean age (*n* = 9) 69.7 years) consented to the research. Six support people had previously participated in aphasia therapy but only three had experience with telerehabilitation services. A total of 372 sessions (63.2% of all delivered sessions) were attended by at least one support person. Support people attended a mean of 64.2% of therapy sessions with their person with aphasia (*SD* = 35.2%; range = 8–98%). Attendance was spread evenly across all therapy types. The support people participated in various therapy activities, including stroke and aphasia education, communication partner training, functional communication practice, and discussion about post-TeleCHAT management plans. Support people provided technical assistance in almost half (49.5%) of the sessions they attended and most frequently during functional and computer therapy. However, two people with aphasia (participants 2 and 3) did not require nor receive any technical assistance from their support people. Support people usually provided technical support on 1–2 tasks per session, such as helping to find and use videoconferencing functions (e.g. annotation tools and remote control), positioning equipment (e.g. setup of a second camera), connecting to the internet, navigating the device (e.g. looking through photos on the iPad), or joining the Zoom meeting.

## Discussion

This is the first study to report the feasible delivery of an ICAP via telerehabilitation. Using a telerehabilitation system purposefully configured to meet user needs and therapy programme requirements, TeleCHAT delivered a high dose of comprehensive aphasia therapy to a heterogeneous group of people with aphasia and their support people. The impairment, functional, and computer-based therapy and psychosocial education sessions delivered in one-on-one and group formats were tailored to participant goals spanning domains of language and functioning. Careful system configuration and tailored training minimised technical issues and user difficulties, enabling the delivery of full or close to the intended 50-h therapy dose to all participants.

This study provides evidence to support the accessibility of and participation in TeleCHAT for a heterogeneous population. Twelve people with aphasia across three cohorts, with varying severities of language impairments, co-morbid conditions, ages and levels of technological confidence, located across metropolitan, rural, and remote locations, successfully completed TeleCHAT. The diversity of participants in this study demonstrates that eligibility to participate in telerehabilitation should not be restricted by any of these factors. These findings challenge perceptions that people with severe aphasia or physical disability are not good candidates for ICAPs,^9^ that assessing participant telerehabilitation readiness for an intensive, comprehensive programme is difficult,^3^ that older adults face significant challenges with using videoconferencing software,^28^ and that people living in remote areas may have slow or insufficient internet speeds to access telerehabilitation.^29^ To facilitate the inclusion of a heterogeneous group and enable delivery of an ICAP to a diverse participant group, speech-language pathologists were trained to understand and consider each person with aphasia's unique requirements to use technology and the technology features that would enable delivery of their planned therapy.^14^ This planning and preparation enabled the speech-language pathologists to provide appropriate support for the person with aphasia to complete the TeleCHAT programme, thereby refuting concerns that the treatment model could not be adapted to support individuals with complex impairments across multiple-language domains.

Nine of the 12 participants received the intended 50 h of therapy, with the remaining three receiving over 88% of the intended dose, demonstrating that it is feasible to deliver high-dose aphasia therapy via telerehabilitation. Concerns regarding the capacity to schedule multiple telerehabilitation sessions^3^ did not arise, with 98.5% of TeleCHAT sessions successfully delivered and 75% of cancellations rescheduled and subsequently delivered. Technical or user issues minimally impacted therapy, with only three sessions cancelled due to technology issues and a high proportion of session time spent actively engaged in therapeutic activities (median session density = 98%). It is likely that the extensive tailoring of the technology to people with aphasia's treatment goals, comorbidities, and technological skills and confidence, as well as, their planned therapy activities, may have reduced user issues. These findings alleviate concerns or perceptions that patient fatigue^30,31^ and internet connection issues impact the feasibility of delivering telerehabilitation.^32^

The versatility of the telerehabilitation system enabled the delivery of 42 distinct goal-directed aphasia therapy activities. The treating speech-language pathologists received explicit training on translating in-person activities for delivery via telerehabilitation^14^ and as such, all planned therapy approaches were successfully delivered. The feasibility of delivering a comprehensive range of therapy approaches is significant as most therapy activities delivered in TeleCHAT had not previously been evaluated or reported in the telerehabilitation literature. Consequently, this study advances our understanding of the feasible delivery of a broad range of aphasia therapies via telerehabilitation. It supports the need for a considered task analysis process as outlined in Vuong et al.^14^

The TeleCHAT programme supported a high proportion of active engagement by support people, not only as technical support but also as participants in education and therapy sessions. The need for technical assistance varied between people with aphasia, with some (*n* = 2) not requiring any aid while others required full assistance. Thus, technical skills and support needs must be assessed and accommodated individually as recommended in Vuong et al.^14^ For those requiring technical assistance, support people provided help in 49.5% of sessions attended (31.3% of all conducted sessions), suggesting that people with aphasia can participate in over half of the telerehabilitation sessions independently if provided with adequate training and opportunity for repeated practice of their technological skills.

Remote delivery of TeleCHAT enabled support people who were not living with the person with aphasia, to participate in group therapy, education, and communication partner training, aligning with aphasia best practice and stroke clinical guidelines.^33,34^ Consequently, all participants had at least one support person join sessions, and over 63% of therapy sessions involved support people. This outcome is important as aphasia literature has reported environmental barriers to conducting family education and communication partner training in-person due to logistical barriers, such as the distance from service location, work commitments, and timing restraints.^9,11,35,36^

Several study limitations were identified which should be addressed in future research. Firstly, selection bias may be present as people with aphasia self-nominated to participate and may have been intrinsically motivated to complete the programme despite potentially encountering issues such as fatigue. However, this recruitment method reflects clinical practice, where patients likely self-nominate their participation in telerehabilitation. Selection bias may be addressed in a future randomised controlled study comparing in-person and telerehabilitation delivery of the intervention. Secondly, feasibility of recruitment was not collected as it was not an objective of this preliminary study, conducted in a research setting. Recruitment feasibility would be appropriate to evaluate in a future implementation trial that determines the service feasibility and cost-effectiveness in diverse clinical settings. Thirdly, indirect time that speech-language pathologists spent troubleshooting technology issues outside of session time was not collected. Future research should investigate the work responsibilities of speech-language pathologists, including direct and indirect clinical tasks. Lastly, personal bias may have been introduced by the first author as she developed the TeleCHAT training package, delivered TeleCHAT and collected data. While a second speech-language pathologist co-delivered the therapy and collected data, and the broader research team reviewed the data analysis, future research designs could further reduce potential bias by recruiting independent speech-language pathologists to deliver TeleCHAT, and blinding outcome assessors.

This study marks a pivotal advancement in aphasia rehabilitation and telerehabilitation by confirming for the first time the feasible delivery of an ICAP via telerehabilitation. Individualised preparation, training, technological tailoring, and goal-matched treatment planning were key to ensuring all participants could participate in TeleCHAT regardless of their aphasia severity or baseline technical skills and receive an intensive dose of comprehensive therapy. In order to ensure resilience in healthcare services during crises such as the COVID-19 pandemic and natural disasters, where telerehabilitation services may be the only available therapy option, it is important to continue future research and advancement of this service delivery model. Future studies may explore the usability of the system, and the acceptability of the programme to its end-users. A phase II randomised controlled trial could also determine clinical efficacy of this programme. The TeleCHAT programme presents an innovative model of care that has the potential to increase access to high-quality, comprehensive, and intensive therapy for people with aphasia.

**Clinical messages**

- It was feasible to deliver an intensive comprehensive aphasia programme via telerehabilitation (TeleCHAT) to a heterogeneous group of people with aphasia and support people. Participants should not be excluded from telerehabilitation services due to their aphasia severity, technical skills, or confidence.
- Telerehabilitation delivery did not impact the dose or comprehensiveness of TeleCHAT.
- Individualised preparation including tailored technical setup, training, and goal-matched treatment planning are key for the feasible delivery of aphasia telerehabilitation. Human-centred design and task analysis should be used to tailor technology to all telerehabilitation users.

## Supplemental Material

sj-docx-1-cre-10.1177_02692155251375667 - Supplemental material for Feasibility of delivering TeleCHAT: A comprehensive high-dose aphasia treatment via telerehabilitation Supplemental material, sj-docx-1-cre-10.1177_02692155251375667 for Feasibility of delivering TeleCHAT: A comprehensive high-dose aphasia treatment via telerehabilitation by Genevieve Vuong, Jade Dignam, Clare Burns, David Copland, Hannah Wedley, Katherine O’Brien and Annie J Hill in Clinical Rehabilitation

sj-docx-2-cre-10.1177_02692155251375667 - Supplemental material for Feasibility of delivering TeleCHAT: A comprehensive high-dose aphasia treatment via telerehabilitation Supplemental material, sj-docx-2-cre-10.1177_02692155251375667 for Feasibility of delivering TeleCHAT: A comprehensive high-dose aphasia treatment via telerehabilitation by Genevieve Vuong, Jade Dignam, Clare Burns, David Copland, Hannah Wedley, Katherine O’Brien and Annie J Hill in Clinical Rehabilitation

sj-docx-3-cre-10.1177_02692155251375667 - Supplemental material for Feasibility of delivering TeleCHAT: A comprehensive high-dose aphasia treatment via telerehabilitation Supplemental material, sj-docx-3-cre-10.1177_02692155251375667 for Feasibility of delivering TeleCHAT: A comprehensive high-dose aphasia treatment via telerehabilitation by Genevieve Vuong, Jade Dignam, Clare Burns, David Copland, Hannah Wedley, Katherine O’Brien and Annie J Hill in Clinical Rehabilitation
