# Supplementary material for: Feasibility of delivering TeleCHAT: A comprehensive high-dose aphasia treatment via telerehabilitation
Source: Clin Rehabil. 2025 Sep 26;39(12):1606–22. doi: 10.1177/02692155251375667 (PMC12615849; doi:10.1177/02692155251375667)
Supplement: sj-docx-1-cre-10.1177_02692155251375667 - Supplemental material for Feasibility of delivering TeleCHAT: A comprehensive high-dose aphasia treatment via telerehabilitation [file sj-docx-1-cre-10.1177_02692155251375667.docx]

**The feasibility of delivering TeleCHAT: A Comprehensive High-dose Aphasia Treatment via telerehabilitation.**

**Supplementary Material 1**

Genevieve Vuong^1,2,3,4,5^, Jade Dignam^1,2,4^, Clare L. Burns^2,6^, David Copland^1,2,3,4^, Hannah Wedley^1,4^, Katherine O’Brien^1,4^, Annie J. Hill^1,2,3,4^

Queensland Aphasia Research Centre, The University of Queensland, Australia

School of Health and Rehabilitation Sciences, Faculty of Health and Behavioural Sciences, The University of Queensland, Australia

Centre for Research Excellence in Aphasia Recovery and Rehabilitation, La Trobe University, Melbourne, Australia

Surgical Treatment and Rehabilitation Service (STARS) Education and Research Alliance, The University of Queensland and Metro North Health, Queensland, Australia

Faculty of Health, Southern Cross University, Bilinga, Queensland, Australia

Speech Pathology and Audiology Department, Royal Brisbane and Women’s Hospital, Metro North Health, Queensland, Australia

Correspondence details:

Genevieve Vuong BSpPath (Hons I)
Email: [g.vuong@uq.edu.au](mailto:g.vuong@uq.edu.au)

**
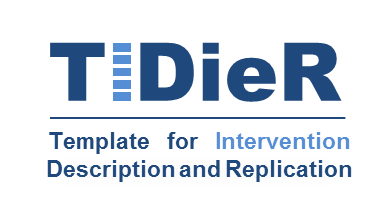
The TIDieR (Template for Intervention Description and Replication) Checklist***

**Modified for reporting of telehealth interventions used in clinical trials (Rhon et al., 2022)**

| **Item** | **Other Information** |
| --- | --- |
| 1. Provide the name or a phrase that describes the intervention  Should include the word “telehealth” (or a term that very clearly indicates that an intervention is being delivered remotely - e.g., “telemedicine, remote digital health, telephone”) in the brief name that describes the intervention. | TeleCHAT – The delivery of the Comprehensive High-dose Aphasia Treatment program via telerehabilitation. |
| 2. Describe any rationale, theory, or goal of the elements essential to the intervention  Provide the rationale for using a telehealth intervention. Is remote delivery used to expand access, or to enhance safety for participants? Is remote delivery an evidence-based option for the intervention? Is the goal to validate use of a traditional in-person intervention delivered in a remote format? | The CHAT program is an evidence-based intervention delivered as part of a health service in QLD, Australia (Dignam et al., 2023). However, it required in-person visits up to three days a week, which posed as an access barrier for people who were geographically distanced and/or had medical mobility and transportation difficulties. The aim of this feasibility trial was to evaluate a telerehabilitation model for the delivery of CHAT, referred to as TeleCHAT.  See also *Introduction* section of manuscript |
| 3. Materials: Describe any physical or informational materials used in the intervention, including those provided to participants or used in intervention delivery or in the training of intervention providers. Provide information on where the materials can be accessed (e.g. online appendix, URL).  What components does the telehealth intervention include (e.g., audio, video, name of platform or software)? What (if any) additional documentation, instruction and/or equipment was provided (loaned or given) to participants? Were participants that did not have access to the equipment or platform excluded? | The TeleCHAT program was delivered via the videoconferencing software, Zoom© (enterprise plan). The university’s enterprise plan of Zoom offered end-to-end encryption at a minimum key strength of 2048-bit encryption and was secured by a minimum Transport Layer Security Version 1.2. Zoom was downloaded onto participants’ device(s). To ensure privacy, Zoom functions such as camera and microphone settings, screensharing, remote control and recording were only enabled with the participant’s consent, meaning that no function was set to perform automatically.  Speech-language pathologists (SLPs) primarily delivered TeleCHAT from a telerehabilitation suite, located in university spaces at a tertiary hospital and health service. The telerehabilitation suite was soundproof, had a computer connected to the university’s internet, two desktop monitors, an external web camera, document camera, iPad, keyboard and mouse. When it was necessary to work from home, clinicians delivered TeleCHAT from a Dell Laptop with an inbuilt web camera, connected to a second desktop monitor. SLPs used their home broadband internet, and/or an internet dongle.  People with aphasia (PWA) participated in TeleCHAT using their personal devices where appropriate, which included MacBooks, Windows laptops, iPads, Mac Computers. Some PWA were required to screenshare from additional personal technology including iOs and android smartphones, ipads, and android tablets. Some PWA needed to wear a headset, have an external web camera, or use a stylus. PWA used their own internet plans, which varied between participants. Where PWA did not have the necessary technology (e.g. ipads, headsets, web cameras, apple pens, internet dongles), this was lent to them by the Queensland Aphasia Research Centre’s Aphasia Tech Hub, The University of Queensland.  Technology Training manuals created by the research team were provided to PWA and SLPs. These manuals included quick reference guides for using technology, therapy session setups and troubleshooting technology.  Resources developed for the delivery of the program included protocols and standard procedures, including risk assessments for delivering the program, digitised therapy materials, and in-session troubleshooting and support resources for SLPs and PWAs. For PWAs, the resources were presented in an aphasia-friendly format and explained in detail before commencing the program. At the start of each session, the SLP completed a ‘Within Therapy Checklist’, adapted from Øra, Kirmess, Brady, Sørli, et al. (2020), which prompted them to confirm that the person with aphasia was prepared and motivated for the session, and identify and address any patient-related issues.  For SLPs to use within therapy sessions, the research team created a therapy checklist, and a progress note template and error registration form which was modified from Ora et al. (2020).  Interactive digital templates were tailored using personal photos and/or pictures of core words. The digital resources for each therapy type is listed below:   - Impairment Therapy   - Powerpoint/PDF templates for therapy activities e.g., Semantic Feature Analysis and Phonological Components Analysis, Repetition in Presence of a picture, Novel Approach to Real-life communication: Narrative Intervention in Aphasia. - Functional Therapy   - Compensatory technology using smart device applications (E.g., voice-text functions, digital picture books)   - Microsoft Office suite to create personalised scripts   - Web browser to search for relevant images and videos - Computer Therapy   - Aphasia therapy software applications available on the iPad (e.g., Tactus Therapy^©^, Constant Therapy^©^)   - Aphasia therapy software applications available on android tablets (e.g., ListenIn^©^)   - Aphasia therapy software programs available on a computer (e.g., Step by Step^©^ therapy, Aphasia Scripts^©^) - Group Therapy   - PowerPoint presentations on topics to facilitate aphasia and stroke education. |
| 4. Procedures: Describe each of the procedures, activities, and/or processes used in the intervention, including any enabling or support activities.  Were the procedures for this intervention originally developed for in-person or remote delivery (number of minutes, open accessibility or requires sign-up/set-up)? What, if anything, was done to adapt procedures from in-person delivery? | The TeleCHAT protocol was devised from the Comprehensive High-Dose Aphasia Therapy program, a 50-hour aphasia therapy program spread over eight weeks, which in previous research, was delivered in-person (Dignam et al., 2023)  The following adaptions were made to enable remote delivery of the intervention.   - Telerehabilitation training for the PWA was provided in the week before they commenced therapy and was provided in-person and/or via telepractice. - Clinical planning involved consideration of technology needed to achieve therapy tasks and goals for each patient. - Assessment was administered either in-person or via telepractice. - PWA were required to sign up for a free Zoom^©^ account if they did not already have one. The SLP then added the PWA as a Zoom^©^ contact. - To begin therapy sessions, PWA were required to open the Zoom^©^ application and wait for the SLP to directly video-call them. PWA then accepted the call and allowed video recording as per prompts. If the SLP could not video-call the PWA, the SLP would call the PWA on their phone to troubleshoot. As a backup, a recurring meeting link and meeting ID number were provided to each participant. - A virtual waiting room was enabled by the treating SLP. The PWA would wait in the waiting room until the SLP was ready to begin the session. - Therapy resources were digitised (See *Materials*) - Where the participant was not able to perform a physical task (e.g., physical set up of a device) or had difficulty troubleshooting technology, a support person (e.g., family member) was required to assist, verbally guided by the SLP. - For therapy tasks (e.g., writing therapy or computer therapy) where physical resources could only be used on one participant’s end (i.e., PWA or SLP end), screensharing functionality (with a secondary camera where necessary) was used to allow the other participant to see the therapy resource. - For therapy tasks (e.g., Semantic feature analysis, Treatment of Underlying forms) where therapy stimulus needed to be interacted with (e.g., pointed to, moved, drawn on, typed on) remote control functionality and/or annotation functionality were used as needed. - Telerehabilitation Training for SLPs.   Training delivered was the same as described in Vuong et al., 2024. The treating speech-language pathologists participated in four hours of CHAT training, which included education regarding the core components of CHAT, clinical case studies and adherence to treatment fidelity. Additional self-access online material was provided, including the CHAT treatment protocol, summaries and videos of treatment procedures, and links to the research literature. The SLP received six hours of telerehabilitation and technology training, delivered across self-access online modules and a practical session involving a simulated therapy session. This training incorporated instruction on completing a task analysis to translate therapy activities for delivery via telerehabilitation, setting up and operating Zoom for TeleCHAT, and strategies to build rapport, mitigate risks, and troubleshoot technical difficulties.   - Telerehabilitation training for PWA and SP:   In the first cohort, training was delivered during in-person home visits and in conjunction with assessment and goal setting as described in Vuong et al., 2024. In cohorts 2 and 3, training was conducted after completing assessments and goal setting, in the week immediately before therapy. Training included a mock telerehabilitation activity, whereby one SLP facilitated a telerehabilitation session from the person with aphasia’s home with the other speech-language pathologist located at QARC. Ten participants were provided training in person. Two participants received all telerehabilitation training via Zoom due to COVID-19 physical distancing restrictions. These participants received support from a support people (in person) during the mock telerehabilitation session with the remote speech-language pathologist.  The range of therapy activities that were delivered during TeleCHAT were similar to those delivered in CHAT (Dignam et al., 2023) and is also listed in Table 5 and Supplementary Table 4. |
| 5. For each category of intervention provider (e.g. psychologist, nursing assistant), describe their expertise, background and any specific training given.  Who delivered the telehealth intervention? Was there any training that went into the delivery of the intervention? Who all was authorized/approved to deliver it and how did they achieve authorization approval (e.g., training, certification process)? | Two qualified, certified practicing SLPs delivered the TeleCHAT intervention to three cohorts. They were required to have graduate experience in aphasia rehabilitation. The SLPs received telerehabilitation training prior to delivery of the program. The telerehabilitation training was developed by the TeleCHAT research team (*See Training and support for fidelity in the manuscript).* |
| 6. Describe the modes of delivery (e.g., face-to-face or by some other mechanism, such as internet or telephone) of the intervention and whether it was provided individually or in a group.  Indicate whether the intervention was delivered solely through remote methods or in a hybrid (remote + in-person) format. Synchronous versus asynchronous, unidirectional or bidirectional (could the participant/attendee ask questions, respond, interact and if so how - voice, chat, etc.?) | Assessment and training were conducted by the treating SLPs prior to the therapy commencement. 8 participants were assessed in-person, 11 were assessed in person and via telerehabilitation, and 1 person was assessed completely via telerehabilitation. 11 participants received training in-person, and 1 participant completed training via telerehabilitation. The TeleCHAT therapy program was delivered solely via synchronous, bidirectional telerehabilitation, individually to PWA and in group settings. |
| 7. Describe the type(s) of location(s) where the intervention occurred, including any necessary infrastructure or relevant features.  Were clinicians in the clinic or at their home? Were patients in the clinic, another remote clinic, or at home? | The PWA received therapy from their homes (residence at the time of therapy, including support person’s home or usual home). The PWA’s home required internet connection and a private, quiet room. They also needed access to their technology devices (see *Materials*).  The SLPs delivered therapy from a telerehabilitation suite at The University of Queensland. When it was necessary to work from home, SLPs delivered TeleCHAT from work devices, using home broadband internet, and/or an internet dongle. See *Materials* for list of infrastructure setup. |
| 8. Describe the number of times the intervention was delivered and over what period of time including the number of sessions, their schedule, and their duration, intensity or dose.  Provide the planned intervention dosing (visits, frequency, duration, etc.) for the trial (expected treatment to meet optimal fidelity) and then also the number of actual visits received. Provide duration and frequency of sessions. | The planned dose to be delivered in TeleCHAT was 50 hours of therapy across 8 weeks of therapy, delivered in 1-2 sessions/therapy day, 3-4 days per week.  A summary of the dose received by participants is reported using the MDAF, in Tables 2 and 3.  Overall, most participants (n = 11) attended 8 weeks of therapy. They attended a median of 1 – 2 sessions/therapy day for 4 days per week. Most participants’ (*n* = 9) sum session length reached the intended 50-hour dose. A high proportion of therapy sessions were spent actively engaged in therapy activities (range = 94 - 100%). Only one participant received a reduced session length (44 hours 13 minutes) as a natural disaster prevented their participation in one week (7 hours) of therapy.  See also *Results* in the manuscript. |
| 9. If the intervention was planned to be personalized, titrated or adapted, then describe what, why, when, and how.  Describe the flexibility of the intervention to allow for any changes in or tailoring of the telehealth intervention for specific patients or groups. | The TeleCHAT program was tailored and personalised to each PWA. Actions taken to tailor therapy included:   - PWAs and their support person participated in collaborative goal setting to ensure that their goals were highly salient and relevant. - From the goals, SLPs and the research team created a tailored therapy plan, matching evidence-based therapy activities to PWA’s goals. - SLPs and the research team used the clinical assessment results to tailor therapy to the PWA’s appropriate level of difficulty - Through a task analysis, the research team identified the optimal technology set-up to deliver the PWA’s therapy plan. - PWAs identified personalised target items, photos, and videos for therapy, and sent these to the SLPs to create therapy resources. - PWA received therapy on their personal devices where possible. - SLPs also personalised therapy schedules for each PWA, which considered their fatigue levels at different times of the day, time zone differences, and other commitments. |
| 10. If the intervention was modified during the course of the study, describe the changes (what, why, when, and how).  If it was planned remotely or in-person and then had to be switched to the other, provide the timing, reasons, and rationale for the change.  If it was planned remotely or in-person and then had to be switched to the other, provide the timing, reasons, and rationale for the change. | For the first cohort of participants, the telerehabilitation training was delivered over 2-3 sessions within the four weeks prior to therapy commencing. However, after feedback from PWAs and SLPs, the training was moved to occur within the week prior to commencement of therapy. The practical component was modified into a mock therapy session facilitated by two SLPs – one located with the PWA, and one located in the QARC tele-suite.  The training, clinical assessment and research measures pre-therapy were administered in-person for most participants. However, for those who were geographically distanced (in different state), these assessments and measures were delivered via telerehabilitation.  Participants’ communication goals and therapy plans were continually revised as they progressed through the TeleCHAT program. Consequently, additional therapy activities were introduced throughout the program, or therapy activities increased in complexity, hence requiring the use of different technology configurations or technological functions. |
| 11. Planned: If intervention adherence or fidelity was assessed, describe how and by whom, and if any strategies were used to maintain or improve fidelity, describe them.  Identify any specific strategies used to improve adherence to the telehealth intervention. Was there a plan to monitor and track fidelity of the intervention? | The following processes were taken to maintain fidelity of the program:   1. Comprehensive TeleCHAT training package for speech pathologists (including access to comprehensive CHAT manual, video demonstrations, practical telerehabilitation training) 2. Clinical Planning sessions held with the research team to plan appropriate, tailored therapy and technology configuration for each PWA. 3. Weekly debrief meetings held by the two treating SLPS to discuss therapy progress of each participant as well as any technological, service and patient related issues and how to resolve them. These meetings were also used to co-plan therapy for the next week. 4. Clinical meetings with the research team (ad-hoc and scheduled mid-way review) to discuss patient progress clinical problem and technical issue resolution. 5. Video-recording of all sessions. SLPs reviewed recordings to reflect on and compare treatment delivery. Research team reviewed recordings where necessary to assist with planning. 6. Patients’ adherence to the telerehabilitation was documented in progress notes which detailed attendance, session length, and length of inactive episodes (time taken to resolve technical issues and user difficulties). 7. SLPs completed a ‘within-therapy checklist’ at the start of each session to ensure all patients were appropriately set up and equipment was working for the telerehabilitation session. 8. An audit of clinical documentation, including technical issues and user difficulties experienced was completed prior to a mid-way evaluation, and at the end of each block of intervention. Results of this audit was discussed between the research team and the clinicians, to improve adherence to the intervention.   See also *Baseline Assessment, Goal Setting, Planning and Training* section of the manuscript. |
| 12. Actual: If intervention adherence or fidelity was assessed, describe the extent to which the intervention was delivered as planned.  Telehealth Addition  Did the telehealth intervention influence actual treatment adherence? Was the fidelity of the telehealth intervention reported? | The extent to which TeleCHAT was delivered as planned is reported in the *Results* section of this paper. Briefly:   1. All participants completed the program completely via telerehabilitation. Therapy was delivered a median of 1.5 sessions per therapy day, 4 days a week, for 8 weeks. 2. Participant demographics and characteristics were diverse across age, gender, time post stroke onset, aphasia profile, experience and confidence using technology. 3. A wide range of therapy activities were delivered targeting personalised goals across the WHO-ICF. 4. Support people actively participated in therapy sessions including stroke and aphasia education and communication partner training. |
