# Supplementary material for: Feasibility of delivering TeleCHAT: A comprehensive high-dose aphasia treatment via telerehabilitation
Source: Clin Rehabil. 2025 Sep 26;39(12):1606–22. doi: 10.1177/02692155251375667 (PMC12615849; doi:10.1177/02692155251375667)
Supplement: sj-docx-2-cre-10.1177_02692155251375667 - Supplemental material for Feasibility of delivering TeleCHAT: A comprehensive high-dose aphasia treatment via telerehabilitation [file sj-docx-2-cre-10.1177_02692155251375667.docx]

**The feasibility of delivering TeleCHAT: A Comprehensive High-dose Aphasia Treatment via telerehabilitation.**

**Supplementary Material 2-4**

Genevieve Vuong^1,2,3,4,5^, Jade Dignam^1,2,4^, Clare L. Burns^2,6^, David Copland^1,2,3,4^, Hannah Wedley^1,4^, Katherine O’Brien^1,4^, Annie J. Hill^1,2,3,4^

1. Queensland Aphasia Research Centre, The University of Queensland, Australia
2. School of Health and Rehabilitation Sciences, Faculty of Health and Behavioural Sciences, The University of Queensland, Australia
3. Centre for Research Excellence in Aphasia Recovery and Rehabilitation, La Trobe University, Melbourne, Australia
4. Surgical Treatment and Rehabilitation Service (STARS) Education and Research Alliance, The University of Queensland and Metro North Health, Queensland, Australia
5. Faculty of Health, Southern Cross University, Bilinga, Queensland, Australia
6. Speech Pathology and Audiology Department, Royal Brisbane and Women’s Hospital, Metro North Health, Queensland, Australia

Correspondence details:

Genevieve Vuong
BSpPath (Hons I)
Email: [g.vuong@uq.edu.au](mailto:g.vuong@uq.edu.au)
ORCID: 0000-0003-3776-3899
Twitter: @genevievevuong

**Supplementary Table 2.** *Individual Participant Demographics and Technology Characteristics*

| **PWA** | **1** | **2** | **3** | **4** | **5** | **6** | **7** | **8** | **9** | **10** | **11** | **12** |
| --- | --- | --- | --- | --- | --- | --- | --- | --- | --- | --- | --- | --- |
| Demographics | | | | | | | | | | | | |
| Sex | M | M | F | M | F | M | M | F | M | F | F | M |
| Age (Years) | 64 | 54 | 24 | 73 | 80 | 59 | 66 | 81 | 72 | 67 | 76 | 66 |
| Education (ISCED)^d^ | 6 | 3 | 4 | 4 | 7 | 2 | 3 | 1 | 1 | 4 | 3 | 6 |
| Handedness | R | R | R | R | L | R | R | R | R | R | R | R |
| TPO | 3;5 | 2;0 | 1;9 | 0;7 | 6;4 | 0;10 | 1;0 | 1;3 | 0;4 | 6;6 | 2;7 | 0;4 |
| # Strokes | 1 | 1 | 1 | 2 | 1 | 1 | 1 | 3 | 1 | 1 | 3 | 1 |
| Lesion Hemisphere of stroke | L | L | L | L | R | L | L | L, R | L | L | L | L |
| Comorbidities impacting | Nil | Speech | UL Move-ment | Sight | Nil | Sight | Speech | Sight, UL movement, Speech, Voice | Writing, Speech | Speech | Speech | UL Move-ment |
| Location^a^ | Rural | Metro | Metro | Metro | Metro | Rural | Metro | Metro | Remote | Metro | Rural | Rural |
| Aphasia severity^b^ | 47.67 | 55.67 | 54.17 | 46 | 55.67 | 40.17 | 58 | 51.83 | 40.67 | 54 | 50.17 | 54.17 |
| Technology Characteristics | | | | | | | | | | | | |
| Personal technology devices | Laptop phone | Laptop ipad | Laptop ipad phone | iPad phone | Computer tablet | Laptop tablet | Laptop ipad, iphone | Laptop, ipad | Laptop, ipad, phone | ipad, phone | Computer ipad | Laptop ipad iphone |
| Confidence using technology^c^ | 3 | 3 | 5 | 2 | 1 | 2 | 4 | 1 | 1 | 4 | 4 | 2 |

*Note*. PWA = Participant with Aphasia. ISCED = International Standard Classification of Education. TPO = Time Post-stroke-Onset (in Years; Months). UL = Upper Limb. # = number of. SP = Support Person. a = According to Rural, Remote and Metropolitan Area (RRMA) classification. b = Average total of six tests on the Comprehensive Aphasia Test T-Scores. c = rating on 5-point scale with 1= Definitely no – 5 = Definitely, yes. d = 1: Primary, 2: Lower secondary 3: Upper secondary, 4: Post-secondary non-tertiary, 5: Short cycle tertiary, 6: Bachelor’s or equivalent, 7: Master’s or equivalent level.

**Supplementary Table 3.** *Individual Participant Aphasia Profiles*

| **PWA** | **1** | **2** | **3** | **4** | **5** | **6** | **7** | **8** | **9** | **10** | **11** | **12** |
| --- | --- | --- | --- | --- | --- | --- | --- | --- | --- | --- | --- | --- |
| Aphasia severity^a^ | 47.67 | 55.67 | 54.17 | 46 | 55.67 | 40.17 | 58 | 51.83 | 40.67 | 54 | 50.17 | 54.17 |
| Auditory Compre-hension. (>56) | 39 | 57^^^ | 52 | 49 | 49 | 42 | 53 | 51 | 34 | 55 | 52 | 58^^^ |
| Reading Compre-hension. (>59) | 41 | 56 | 53 | 49 | 51 | 38 | 54 | 46 | 46 | 50 | 53 | 51 |
| Repetition (>59) | 48 | 52 | 53 | 45 | 52 | 41 | 62^^^ | 53 | 34 | 53 | 47 | 52 |
| Naming (>62) | 50 | 60 | 58 | 46 | 60 | 35 | 59 | 59 | 40 | 54 | 44 | 55 |
| Reading Aloud (>60) | 57 | 51 | 54 | 44 | 61^^^ | 38 | 61^^^ | 54 | 44 | 52 | 43 | 49 |
| Writing (>57) | 51 | 58^^^ | 55 | 43 | 61^^^ | 47 | 59^^^ | 48 | 46 | 60^^^ | 62^^^ | 60^^^ |

*Note*. a = Average of six modality means on the Comprehensive Aphasia Test T-Scores. ^ = T-Score is above cut-off. Cut-offs are stated in brackets next to the language domain.

**Supplementary Table 4.** *Individual Participant Dose per Therapy Type*

|  | **Impairment** | | | | **Computer** | | | | | **Functional** | | | | | | **Group** | | | |
| --- | --- | --- | --- | --- | --- | --- | --- | --- | --- | --- | --- | --- | --- | --- | --- | --- | --- | --- | --- |
| **PWA** | **Session Length** | **Inactive Episodes** | **Active Episodes** | **Session Density** | | **Session Length** | **Inactive Episodes** | **Active Episodes** | **Session Density** | | **Session Length** | **Inactive Episodes** | **Active Episodes** | **Session Density** | **Session Length** | | **Inactive Episodes** | **Active Episodes** | **Session Density** |
| 1 | 14:46 | 0:05 | 14:41 | 99 | | 14:53 | 0:20 | 14:33 | 98 | | 14:52 | 0:28 | 14:24 | 97 | 8:20 | | 0:01 | 8:19 | 100 |
| 2 | 14:58 | 0:07 | 14:51 | 99 | | 14:46 | 0:16 | 14:30 | 98 | | 14:41 | 0:04 | 14:37 | 100 | 8:20 | | 0:07 | 8:12 | 98 |
| 3 | 13:25 | 0:00 | 13:25 | 100 | | 12:52 | 0:08 | 12:43 | 99 | | 14:06 | 0:00 | 14:06 | 100 | 8:20 | | 0:00 | 8:20 | 100 |
| 4 | 14:26 | 0:30 | 13:56 | 97 | | 15:16 | 1:19 | 13:57 | 91 | | 14:52 | 0:54 | 13:58 | 94 | 8:13 | | 0:29 | 7:44 | 94 |
| 5 | 14:48 | 0:22 | 14:26 | 98 | | 14:37 | 0:46 | 13:51 | 95 | | 14:55 | 0:58 | 13:57 | 94 | 8:29 | | 0:19 | 8:10 | 96 |
| 6 | 14:17 | 0:00 | 14:17 | 100 | | 14:12 | 0:13 | 13:59 | 98 | | 14:33 | 0:12 | 14:20 | 99 | 8:33 | | 0:17 | 8:16 | 97 |
| 7 | 13:49 | 0:28 | 13:21 | 97 | | 14:13 | 1:51 | 12:21 | 87 | | 13:51 | 0:31 | 13:20 | 96 | 7:24 | | 0:19 | 7:05 | 96 |
| 8 | 15:00 | 0:41 | 14:19 | 95 | | 14:23 | 0:39 | 13:43 | 95 | | 14:37 | 1:06 | 13:31 | 92 | 7:22 | | 0:17 | 7:05 | 96 |
| 9 | 14:20 | 0:12 | 14:07 | 98 | | 14:17 | 0:22 | 13:55 | 97 | | 14:17 | 0:04 | 14:13 | 100 | 8:20 | | 0:00 | 8:20 | 100 |
| 10 | 14:16 | 0:15 | 14:01 | 98 | | 14:34 | 0:25 | 14:09 | 97 | | 14:24 | 0:27 | 13:57 | 97 | 8:17 | | 0:00 | 8:17 | 100 |
| 11 | 13:14 | 0:40 | 12:34 | 95 | | 11:00 | 0:05 | 10:55 | 99 | | 13:39 | 0:44 | 12:55 | 95 | 6:20 | | 0:00 | 6:20 | 100 |
| 12 | 14:26 | 0:21 | 14:05 | 98 | | 14:31 | 0:20 | 14:11 | 98 | | 14:36 | 0:25 | 14:11 | 97 | 8:18 | | 0:00 | 8:18 | 100 |
| Total | 171:45 | 3:41 | 168:03 | 98 | | 169:34 | 6:45 | 162:48 | 96 | | 173:23 | 5:53 | 167:29 | 97 | 96:16 | | 1:49 | 94:26 | 98 |
| *M* | 14:18 | 0:18 | 14:00 | 98 | | 14:07 | 0:33 | 13:34 | 96 | | 14:26 | 0:29 | 13:57 | 97 | 8:01 | | 0:09 | 7:52 | 98 |
| SD | 0:52 | 0:14 | 0:37 | 71 | | 1:35 | 0:31 | 1:03 | 66 | | 0:51 | 0:22 | 0:29 | 57 | 0:50 | | 0:10 | 0:40 | 80 |
| Range | 12:34 -15:32 | 0:00- 0:41 | 12:34 -14:51 | 95.0 -99.4 | | 11:00 -16:24 | 0:05 - 1:51 | 10:55 -14:33 | 86.9 -99.2 | | 12:55 -15:43 | 0:00 - 1:06 | 12:55 -14:37 | 92.5 -100 | 6:20 - 8:49 | | 0:00 - 0:29 | 6:20 - 8:20 | 94.1-100 |

*Note.* PWA = Participant with Aphasia. SL = Session Length (hours and minutes). IE = Inactive Episodes (hours and minutes). AE = Active Episodes (hours and minutes). SDen = Session Density (%). *M* = Mean. *SD* = Standard Deviation.

**Supplementary Table 5.** *Frequency Count of All Therapy Activities Delivered to Each Participant.*

| **Participant No.** | **1** | **2** | **3** | **4** | **5** | **6** | **7** | **8** | **9** | **10** | **11** | **12** | **Grand Total** |
| --- | --- | --- | --- | --- | --- | --- | --- | --- | --- | --- | --- | --- | --- |
| Impairment Therapy | | | | | | | | | | | | | |
| A structured approach to text messaging | - | - | - | - | - | - | - | - | - | 13 | - | - | 13 |
| CART spelling | - | - | - | - | - | - | - | 19 | - | - | - | - | 19 |
| Interactive spelling treatment approach | - | - | - | - | - | - | - | 4 | - | - | - | - | 4 |
| SFA/PCA | 13 | 10 | - | 14 | 9 | 14 | - | 14 | 12 | 14 | 12 | 13 | 125 |
| RIPP | 12 | 8 | - | 14 | - | 2 | - | - | - | - | - | 4 | 40 |
| RISP | - | - | - | - | - | - | 13 | - | - | - | - | 9 | 22 |
| Mapping Therapy | 9 | - | - | - | 4 | - | - | - | - | 11 | - | 10 | 34 |
| TUF | - | - | 9 | - | 4 | - | - | - | - | - | - | - | 13 |
| VNEST | - | 3 | - | - | 6 | - | 12 | 8 | - | - | - | - | 29 |
| NARNIA | - | 4 | 7 | - | - | - | - | - | - | - | 4 | 5 | 20 |
| ARCS reading | - | 2 | - | - | - | - | - | - | - | - | 2 | - | 4 |
| Functional Therapy | | | | | | | | | | | | | |
| Communication Partner Training | 6 | 2 | 1 | 1 | 3 | 2 | 3 | 6 | 5 | 1 | 2 | 3 | 35 |
| Communication repair strategies | - | 2 | - | 1 | 7 | - | 2 | - | 1 | 2 | 2 | 2 | 19 |
| Functional/Metacognitive strategies phone calling | - | - | - | - | - | - | - | - | - | 2 | - | 4 | 6 |
| Stroke and Aphasia Education | - | - | 1 | - | 1 | - | 2 | - | - | - | - | - | 4 |
| Compensatory strategies using assistive technology | 3 | 4 | 3 | 3 | - | 1 | 3 | 2 | 3 | 2 | 1 | 6 | 31 |
| Aphasia friendly modifications for technology use | - | - | - | 1 | - | 3 | 2 | 1 | 2 | 5 | - | - | 14 |
| Functional writing/texting | - | - | - | - | - | - | 4 | 10 | - | 5 | - | 6 | 25 |
| Environmental compensatory strategies | - | - | - | - | 3 | - | - | - | - | - | - | - | 3 |
| General counselling | - | - | - | - | 2 | - | 1 | - | - | - | - | - | 3 |
| Training AAC | - | - | - | - | - | - | - | - | 12 | - | - | - | 12 |
| PACE | - | - | - | 8 | - | 9 | - | 1 | - | - | - | - | 18 |
| Training multimodal communication | - | - | - | - | - | - | - | - | 4 | - | 1 | 1 | 6 |
| Personalised Aphasia Cards and Books/Apps | 12 | - | - | 4 | - | 10 | - | - | - | 1 | 1 | - | 28 |
| Role Play | 8 | 9 | 8 | 7 | 4 | 3 | 6 | - | 7 | 2 | 1 | 6 | 61 |
| Script Therapy | 1 | 5 | 6 | 2 | 7 | - | 7 | - | 1 | 4 | 7 | 4 | 44 |
| Real life practice of goal | - | - | 2 | - | - | - | - | - | - | - | - | 2 | 4 |
| Self-management strategies | 3 | 4 | 1 | 5 | 3 | 3 | 4 | 3 | 2 | 2 | 2 | 1 | 33 |
| Linking to other services | 1 | 1 | 2 | 3 | 3 | 4 | 4 | 2 | 1 | 1 | 2 | 2 | 26 |
| Computer Therapy | | | | | | | | | | | | | |
| Aphasia Therapy Online | - | - | - | - | - | 1 | - | - | - | - | - | - | 1 |
| Aphasia Scripts | 4 | 4 | 6 | - | - | - | 1 | - | - | 9 | 3 | - | 27 |
| Constant therapy | 1 | 3 | 8 | 8 | 5 | - | 2 | - | 2 | - | - | 5 | 34 |
| Lingraphica TalkPath Therapy | - | - | - | - | - | 1 | - | - | - | - | 3 | - | 4 |
| Listen In | - | - | - | - | 3 | 1 | - | - | - | - | - | - | 4 |
| Step by step | 12 | 5 | 2 | 13 | - | 14 | - | 14 | 9 | 6 | 6 | 6 | 87 |
| Tactus Advanced therapy | - | 7 | 1 | - | 13 | - | 13 | - | - | - | - | 2 | 36 |
| Tactus Therapy | - | - | - | - | - | - | - | - | 5 | 4 | 3 | 3 | 15 |
| Grand Total | 43 | 43 | 42 | 42 | 43 | 44 | 41 | 44 | 42 | 44 | 37 | 44 | 509 |

*Note*. CART = Copy Action Retell Therapy. SFA/PCA = Semantic Feature Analysis and Phonological Components Analysis. RIPP – Repetition in Presence of a Picture. RISP = Repeated, Increasingly Speeded Production. TUF = Treatment of Underlying Forms. VNeST = Verb Network Strengthening Treatment. NARNIA = Novel Approach to Real-life communication: Narrative Intervention in Aphasia. ARCS = Attentive Reading & Constrained Summarisation. AAC = Augmentative and Alternative Communication. PACE = Promoting Aphasics' Communicative Effectiveness. ASK = Action Success Knowledge program. InterD-CAG = Interdisciplinary Community Aphasia Group.

**Supplementary Table 6.** *Therapy Tasks with Extended Inactive Episodes.*

| **Therapy Type** | **Therapy Activity** | **Causes of issues related to the therapy activity** | **Frequency of therapy activities with extended IE^ (% of times therapy delivered overall)** | **Total IE (min)** | **Mean IE per instance (min)** | **Median IE per instance (min)** | **Range IE per instance (min)** |
| --- | --- | --- | --- | --- | --- | --- | --- |
| Computer | Tactus Advanced Therapy | Screenshare second device, annotation, audio feedback | 9 (25.0%) | 64 | 7 | 8 | 6 - 10 |
| Computer | Constant Therapy* | Poor internet connection when screensharing, screenshare second device, audio feedback, voice recording, annotation | 9 (26.4%) | 44 | 4 | 8 | 8 -20 |
| Functional | Training use of technology to assist daily communication | Setup 2^nd^ camera, joining meeting with correct device, screenshare second device, annotation and remote control | 5 (7.1%) | 42 | 8 | 7 | 6 - 15 |
| Functional | Communication Partner Training | Screenshare second device, navigating device | 1 (2.9%) | 7 | 7 | 7 | 7 - 7 |
| Functional | Training use of AAC | Screenshare, screenshare second device | 1 (3.0%) | 6 | 6 | 6 | 6 - 6 |
| Functional | Script Therapy | Navigating emails, screenshare | 1 (2.3%) | 16 | 16 | 16 | 16 - 16 |
| Impairment | Copy and Recall Treatment | Screenshare second device, setup 2^nd^ camera, 'View' settings, 'Camera' settings, annotation | 2 (10.5%) | 15 | 7 | 7 | 7 - 8 |

*Note.* ^ Criteria for significance is an inactive episode that lasts >=6 minutes (10% or more of the intended therapy time of 60 minutes). * An error in voice recording was experienced only in the Constant Therapy subcategory “Naming”. IE = Inactive Episodes.
