# Supplementary material for: Feasibility of delivering TeleCHAT: A comprehensive high-dose aphasia treatment via telerehabilitation
Source: Clin Rehabil. 2025 Sep 26;39(12):1606–22. doi: 10.1177/02692155251375667 (PMC12615849; doi:10.1177/02692155251375667)
Supplement: sj-docx-3-cre-10.1177_02692155251375667 - Supplemental material for Feasibility of delivering TeleCHAT: A comprehensive high-dose aphasia treatment via telerehabilitation [file sj-docx-3-cre-10.1177_02692155251375667.docx]

Progress Notes

Date: ______________ Session Duration: ____________

Start Time: _____­_____ End Time: __________________

Participant ID: ______________ Clinician ID: ________________

Location of Participant: ___________________ Consent Obtained:  Yes

Did a support person (family member or carer) attend?

Yes  No

Therapy Session:

Impairment  Functional  Computer  Group

Was the intended Session Dose delivered?  Yes  No

Why/Why not? _________________________________________________________________

Devices used:  Desktop  Laptop  iPad  Tablet  Phone

# Within Therapy Checklist

At the beginning of the therapy session, please review the following together with the participant and anyone else in the room.

- Introduction of everybody in the room on both sides
- Clarify security / privacy locally:
  - Is anyone else around that may be able to see or hear the session?
  - Are other people in the participant’s house informed that the therapy session is starting?
- Go through the patient’s checklist:
  - Is the TV / radio nearby turned off?
  - Is the camera adjusted to see the participant (and their carer)?
  - Does the participant have their tablet with them?
  - Does the participant have their phone to call the speech pathologist in case of any technical issues?
- Please ask the patient’s level of motivation for today’s session.

5 – Very motivated for therapy

4 – Somewhat motivated for therapy

3 – Neutral

2 – Not really motivated for therapy

1 – Does not want to do therapy

Reasons (optional): ___________________________________

- A family member needed to help the participant use the technology (review at the end of the session):

To do **all** tasks
 To do **most** tasks
 To do **some** tasks
 Once or twice
 **Did not** help at all
 No family member present

- Speed test:
  - PWA: Download = ; Upload = _________
  - SLP: Download = ; Upload = _________

Session Notes

**On Examination:**

**Goal (#):**

*Impairment Therapy:*

- Activity: (*semantic feature analysis/phonological component analysis, semantic therapy, mapping therapy, treatment of underlying forms, VNest, CILT, RIPP, Narnia, phonological treatment, CART and ACT, syntax treatment, ARCS-W*)
- Target: (*auditory/reading comprehension/spoken language production/spelling/writing at single word, sentence, paragraph and discourse level*)
- Stimuli:
- % accuracy:
- Errors:

Phonemic Paraphasia

Semantic Paraphasia

Neologisms

Grammatical

- Time:
- Cues/prompts used:

Phonemic – Rhyming / Sound / Syllable

Semantic – Function / Sentence Completion

Orthographic – Written /

Modelling

- Notes:

*Functional Therapy:*

- Activity: (*Script therapy, communication-partner training, role play, rehearsal of tasks with/without scripts, use of compensatory strategies, problem solving etc*.)
- Stimuli:
- % accuracy/progress:
- Errors:

Phonemic Paraphasia

Semantic Paraphasia

Neologisms

Grammatical

- Time:
- Cues/prompts used:

Phonemic – Rhyming / Sound / Syllable

Semantic – Function / Sentence Completion

Orthographic – Written /

Modelling

- Notes:

*Computer Based Therapy:*

- Activity: (*Step-by-Step, ORLA, Aphasia Scripts, TACTUS, Constant Therapy, REACT*).
- Stimuli:
- % accuracy:
- Errors:

Phonemic Paraphasia

Semantic Paraphasia

Neologisms

Grammatical

- Time:
- Cues/prompts used:

Phonemic – Rhyming / Sound / Syllable

Semantic – Function / Sentence Completion

Orthographic – Written /

Modelling

- Notes:

*Group Therapy****:***

Session focused on:

- Topic:
- Participation:
- Outcome:
- Notes:

Impressions:

Recommendations/Home Practice Activities:

Plan:

# Error registration

Please complete this log for any technical errors that occur **during** the therapy session.

1. Where did the error occur?

In the speech pathologist´s computer
 In the participant´s computer
 In the internet connection
 In the videoconferencing software
 Unknown
 Other: _______________________________________________________________

1. How did the error occur?

___________________________________________________________________________

1. Was this a technical error or a user error?

Technical error
 User error
 Describe error (optional):_________________________________________

1. What was the consequence of the error?

Delayed session by _______ minutes
 Interrupted session by _______ minutes
 Other consequences: __________________________________________________________________

1. How was the error resolved?

Participant independently resolved

Speech Pathologist guided participant

Speech Pathologist resolved

Tech Support Person contacted

Spontaneous

IT Support
